# Clinical analysis and risk factors associated with poor prognosis in nontuberculous mycobacterial infection

**DOI:** 10.1080/21505594.2025.2459313

**Published:** 2025-02-03

**Authors:** Jinjing Chai, Sujie Zhang, Chong Ma, Qimin Mei, Tao Liu, Jihai Liu, Yecheng Liu, Huadong Zhu

**Affiliations:** a Emergency Department, The State Key Laboratory for Complex, Severe and Rare Diseases, Peking Union Medical College Hospital, Chinese Academy of Medical Sciences and Peking Union Medical College, Beijing, China; b Emergency Department, The First Affiliated Hospital of Zhengzhou University, Zhengzhou, China; c Department of Pulmonary and Critical Care Medicine, Peking Union Medical College Hospital, Peking Union Medical College, Chinese Academy of Medical Sciences, Beijing, China; d Department of health care, Peking Union Medical College Hospital, Peking Union Medical College, Chinese Academy of Medical Sciences, Beijing, China

**Keywords:** Nontuberculous mycobacteria, mortality, comorbidity, risk factors, prognosis

## Abstract

Recently, the incidence and prevalence of NTM have been increasing nationwide in many countries. This study aimed to identify risk factors associated with the prognosis and mortality of non-HIV nontuberculous mycobacterial disease patients. This retrospective study was conducted at Peking Union Medical College Hospital. The electronic medical records in the hospital’s database from January 2013 to December 2022 were retrospectively reviewed. Relevant data, including clinical characteristics, laboratory findings, microbiological tests, treatments, and outcomes were collected and subjected to statistical analyses. The search identified 745 patients diagnosed with NTM infection, of whom 147 met the inclusion criteria. NTM pulmonary disease was the most commonly observed (*n* = 93; 63.3%), followed by disseminated infection (*n* = 43; 29.3%). The most frequent NTM species was *Mycobacterium avium* complex (55.8%), followed by *Mycobacterium abscessus* (21.2%). The incidence of *Aspergillus* and *Pseudomonas aeruginosa* infection was significantly higher in the NTM pulmonary disease group than in the disseminated NTM group. Cumulative mortality in the total patients was 24.49% at 5 years. High Charlson Comorbidity Index (CCI), high neutrophil-to-lymphocyte-ratio (NLR), haematological disease, and disseminated infection were identified as independent predictors of unfavourable outcomes. The area under the curve (AUC) values for NLR and neutrophil-to-monocyte-plus-lymphocyte-ratio (NMLR) were 0.751 and 0.763 with optimal cut-off values of 9.50 and 3.83, respectively, for prediction of mortality in patients with NTM disease.

## Introduction

Nontuberculous mycobacteria (NTM) are recognized as opportunistic pathogens that are difficult to eradicate and require long-term treatment. Recently, the incidence and prevalence of NTM have been increasing nationwide in many countries [1–4], and many clinicians and microbiologists are now paying attention to this disease. Brode et al. [5] showed a simultaneous decrease in tuberculosis (TB) and an increase in NTM disease in many parts of the world. NTM infection has been shown to increase the economic burden on people [6]. In mainland China, the overall prevalence rate of NTM infection among TB suspects was 6.3% [7]. NTM was associated with a high case-fatality rate in people with HIV [8]. The most common clinical presentation of NTM disease is pulmonary disease, but disseminated disease and lymphatic, skin, soft tissue, and bone disease are also important [9]. Disseminated NTM (DNTM) is a rare and refractory infection with no definitive curative therapeutic options that mostly occurs in patients with impaired immunity, especially human immunodeficiency virus/acquired immunodeficiency syndrome (HIV/AIDS) patients with decreased CD4^+^ cell counts [8]. Patients with immune disorders/defects or a history of immunosuppressive therapy are at high risk of DNTM. The associations between immune status and DNTM, clinical characteristics, and mortality in non-HIV patients have not been fully elucidated. It is crucial to clarify the immune status in non-HIV patients to prevent DNTM progression and improve their prognosis. Identifying patients who may recover and patients who may progress or develop serious life-threatening infections would be of huge clinical benefit for doctors to guide treatment decisions [10].

The clinical manifestations of NTM disease involve complex interactions among multiple factors that influence one another, including population susceptibility, NTM species, and host immune status [11]. NTM infection remains a progressive lethal disease associated with high mortality in the absence of timely, effective, and long-time treatment, especially for DNTM. Therefore, it is very important to identify risk factors associated with prognosis and mortality. According to a previous report, the susceptibility to NTM can be increased if the activity of specific T cells is reduced [12]. Old age and male sex are the predictors of mortality in NTM infections, and patients with NTM pulmonary disease caused by *Mycobacterium avium* complex (MAC) had a better prognosis than those caused by rapidly growing NTM (RGM) [13]. Unfortunately, the knowledge of the basic immune status driving susceptibility and the factors linked to poor prognosis of NTM in certain individuals remains limited. Neutrophil-to-lymphocyte-ratio (NLR) and neutrophil-to-monocyte-plus-lymphocyte-ratio (NMLR) are promising and easy-to-obtain valuable biomarkers. A previous study confirmed that posttreatment lymphopenia could predict an increased risk of NTM pulmonary disease redevelopment [14]. Evaluation of these ratios may have the potential to reflect the immune status and predict the prognosis of patients, at least to a certain extent. Few studies have evaluated the prognostic values of these ratios as biomarkers for NTM disease progression. In the present study, we evaluated the clinical characteristics, disease prognosis, cut-off values of the ratios for predicting clinical outcomes, and risk factors associated with mortality in non-HIV patients with NTM disease in China.

## Materials and methods

### Setting and participants

This retrospective study was conducted at Peking Union Medical College Hospital, one of the top tertiary care hospitals in Beijing, China. Patients with NTM were identified for review by searching the hospital’s electronic database using the International Classification of Diseases 10th (ICD-10) codes. The electronic medical records were retrospectively reviewed for all patients with NTM between January 2013 and December 2022 and the following data were collected from identified patients with newly diagnosed NTM disease: (1) demographic characteristics, including age, sex, height, weight, BMI, and smoking status; (2) clinical characteristics, including symptoms, physical signs, imaging examination findings, previous medical history, immunosuppressive medications, comorbidities, prescribed treatments, and outcomes; (3) laboratory findings, including routine blood tests, detection of lymphocyte subsets, C-reactive protein, erythrocyte sedimentation rate, and virus PCR assay results (cytomegalovirus, Epstein-Barr virus, and herpes simplex virus). (4) microbiological tests, including findings for species identification, smears, and cultures of respiratory tract specimens. The study was approved by the Medical Ethics Committee of Peking Union Medical College Hospital (S-K1673). It was carried out by the ethical standards laid down in the 1964 Declaration of Helsinki and its later amendments.

### Inclusion criteria and definitions

The diagnosis of NTM disease was comprehensively judged based on clinical manifestations and radiological, aetiological, and pathological examination findings using the guidelines of the American Thoracic Society/Infectious Diseases Society of America (ATS/IDSA) and British Thoracic Society [9,15,16]. The recommended diagnostic criteria for NTM disease are as follows: (1) DNTM disease: evidence of NTM infection in at least two nonadjacent organ systems, positive NTM culture/molecular tests from the blood, and/or positive NTM culture/molecular tests from bone marrow or a liver biopsy specimen; (2) NTM pulmonary disease: respiratory symptoms, chest radiograph showing cavitary opacities, multifocal bronchiectasis, or multiple small nodule lesions, exclusion of other lung diseases, and microbiological tests performed according to guidelines; (3) extrapulmonary NTM disease: local symptoms, detection of external pulmonary tissue or organ lesions, exclusion of other diseases, positive NTM culture/molecular tests from tissue biopsy or lesion site. RGM and slowly growing NTM (SGM) were defined in accordance with the guidelines [9]. The investigators collected data from the hospital medical records system. Infectious or respiratory experts conducted comprehensive evaluations based on the patients’ condition and determined NTM diseases according to the guidelines [15]. Two experts independently searched the records and conducted a review of the retrieved patients. Subsequently, they cross-checked for the appropriateness of inclusion. Disagreements were resolved through discussion with a third researcher. The date of diagnosis was defined as the first positive culture date [15]. The time to diagnosis was defined as the time between disease onset and the date of diagnosis. The study cohort included all hospitalized patients aged ≥18 years who were newly diagnosed with NTM disease and excluded patients who were diagnosed with NTM disease but did not receive treatment and patients with HIV.

### Microbiological methods

Microbiological laboratory analyses were performed at Peking Union Medical College Hospital. Standard procedures were used to culture and smear sputum acid-fast bacilli [17]. The electronic database of the clinical microbiology laboratory provided positive specimens for the mycobacterium culture. The samples were collected and inoculated into liquid MGIT 960 Mycobacterium Culture Medium (Becton Dickinson UK Ltd., Berkshire, UK) for culture. *Mycobacterium tuberculosis* and NTM were initially differentiated by the MPB64 antigen colloidal gold method. Next, NTM was determined by PCR using the diagnostic kit for *M. tuberculosis*/NTM DNA kit with a PCR-fluorescence probe method (CapitalBio Technology, Chengdu, China). A PCR fluorescent probe method was used to identify clinical specimens of mycobacteria. The method used a combination of double-PCR technology and TaqMan probe technology to qualitatively detect mycobacterial nucleic acids. The species were then identified by microarray hybridization, DNA sequencing, or metagenomic next-generation sequencing (mNGS). We performed *rpoB* DNA sequencing, while DNA sequencing was provided by RuiBiotech Corporation (Beijing, China). The Sanger method was used for the sequencing reactions. After analysis and processing of the sequencing results, the data were imported into NCBI for blast comparison to determine the species information. The procedure of mNGS consisted of nucleic acid extraction, library construction, sequencing, and bioinformatic analysis, as reported in previous research [18]. Pathogens were identified in the samples from the lower respiratory tract (sputum, bronchoalveolar lavage, or bronchial washing), blood, bone marrow, sterile body fluids, pus, or biopsy tissue. In addition to NTM, other pathogens were detected using conventional detection methods, including morphological detection, microbial culture, and pathogen-specific PCR detection, serological pathogen-specific antibody titre detection.

### Statistical analysis

Statistical analyses were performed using SPSS 22.0 software (SPSS Inc., Chicago, IL), and R software version 3.6.1 (R Foundation for Statistical Computing, Beijing, China) was used for survival curve analysis and nomogram creation. The data were tested for a normal distribution by the Kolmogorov – Smirnov test. Nonnormally distributed data were presented as median (interquartile range). Nonnormally distributed variables were compared using the Mann – Whitney U test. The chi-square test or Fisher’s exact test was used for comparisons of categorical variables. Univariate Cox regression analyses were conducted to identify potential risk factors. Relevant factors were subjected to a multivariate Cox analysis using the stepwise selection method. A nomogram was built using the variables and was used to calculate the total scores [19]. We performed a nomogram based on the “rms” R package and applied Cox regression analysis to assess factors related to survival. We selected the prognostic factors to construct the nomogram and used the variables to calculate the total scores of each patient. According to the nomogram, we obtained the total score by adding the values of each variable for predicting the patient’s survival probability. Cut-off values were determined using receiver-operating characteristic (ROC) curves. Values of *p* < 0.05 were considered to indicate statistical significance.

## Results

### Characteristics of the study population

**Figure 1.**
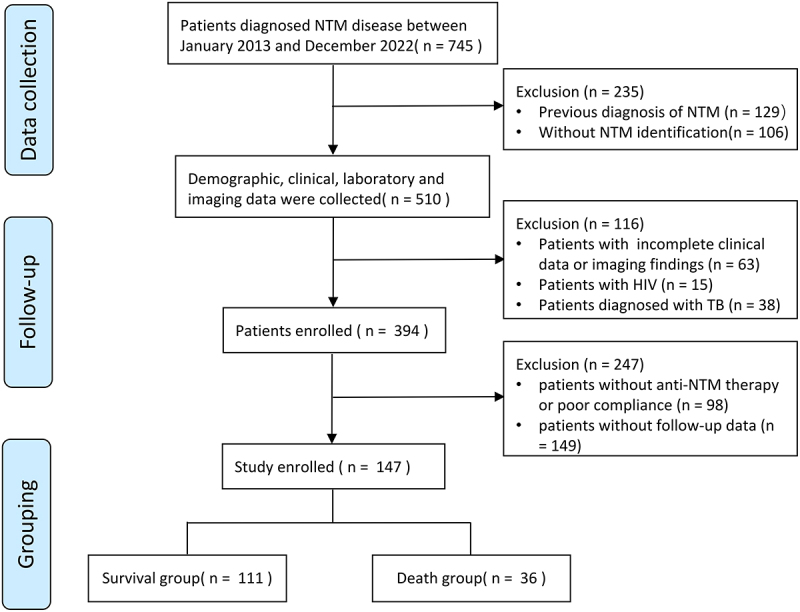
Flowchart for the selection of the study population.

A total of 745 patients diagnosed with NTM disease were screened from the hospital’s medical records system. After excluding these patients who did not meet the inclusion criteria, a total of 147 newly diagnosed NTM disease patients were included in the study (Figure 1). Among these 147 patients, 36 died during the follow-up period, 54% were female, and the median diagnosis time was 13 months. The clinical characteristics, comorbidities, treatments, and prognoses of the 147 patients are shown in Table 1.

**Table 1.** Clinical characteristics of the non-HIV patients with NTM disease.

Laboratory indicators, median [IQR]	Overall (*n* = 147)	Survival (*n* = 111)	Death (*n* = 36)	*P*
Age, y	52(39.0–62.0)	52.0(38.0–62.0)	53.5(41.0–65.0)	0.296
Sex, Female, n (%)	79(53.7)	65(82.3)	14(17.7)	0.040
BMI, median, kg/m^2^	21.0(19.0–23.4)	21.1(19.3–23.4)	20.9(18.0–23.1)	0.642
Smoking, n (%)	52(35.4)	35(31.5)	17(47.2)	0.087
Time to diagnosis(m)	13.0(5.1–35.0)	12.2(4.1–31.7)	14.1(6.3–35.2)	0.469
Comorbidity, n (%)				
CPD	63(42.9)	48(43.2)	15(41.7)	0.868
DM	21(14.3)	16(14.4)	5(13.9)	0.938
AD/CTD	47(32.0)	36(32.4)	11(30.6)	0.834
Chronic liver disease	16(10.9)	13(11.7)	3(8.3)	0.572
CKD	18(12.2)	10(9.0)	8(22.2)	0.036
Immune defect disease	16(10.9)	10(9.0)	6(16.7)	0.200
Solid malignancies	16(10.9)	12(10.8)	4(11.1)	0.960
Haematological disease	19(12.9)	7(6.3)	12(33.3)	0.000
CCI score	2.0(1.0–3.0)	2.0(1.0–3.0)	3.0(2.0–4.0)	0.013
Symptoms at presentation, n (%)				
Cough	106(72.1)	77(69.4)	29(80.6)	0.193
Fever	110(74.8)	78(70.3)	32(88.9)	0.025
Dyspnea	61(41.5)	43(38.7)	18(50.0)	0.233
Fatigue	48(42.1)	34(40.0)	14(48.3)	0.436
Reported weight loss	68(46.3)	50(45.0)	18(50.0)	0.604
Bone pain	32(21.9)	24(21.6)	8(22.9)	0.878
NTM type at diagnosis, n (%)				
MAC	71(48.3)	54(48.6)	17(47.2)	0.546
*M. kansasii*	9(6.1)	5(4.5)	4(11.1)	–
*M. gordonae*	4(2.7)	4(3.6)	0(0)	–
*M. abscessus*	31(21.1)	24(21.6)	7(19.4)	–
*M. fortuitum*	6(4.1)	5(4.5)	1(2.8)	–
Multiple	17(11.5)	12(10.8)	5(13.8)	–
Others	9(6.1)	7(6.3)	2(5.5)	–
Glucocorticoids or immunosuppressive therapy	80(54.4)	51(45.9)	29(80.6)	0.000

NTM, nontuberculous mycobacteria; BMI, body mass index; CPD, chronic pulmonary disease; AD, autoimmune diseases; CTD, connective tissue disorder; CKD, Chronic kidney disease; DM, diabetes mellitus; IQR, interquartile range; MAC, *Mycobacterium avium* complex; CCI score, Charlson Comorbidity Index; HIV, human immunodeficiency virus.

We categorized patients with NTM disease according to their organ involvement and species identification. NTM pulmonary disease was the most commonly observed (*n* = 93; 63.3%), followed by DNTM at the time of diagnosis (*n* = 43; 29.3%). The most frequently detected NTM species was MAC (55.8%), followed by *Mycobacterium abscessus* (21.2%). We found two cases of *Mycobacterium marseillense*, one case of *Mycobacterium chimaera*, and one case of *M. marseillense* and *Mycobacterium simiae* coinfection, both of which presented with disseminated infections. Three of these patients died during follow-up. The other cases were found to be coinfected with two or more types of NTM species. The distribution of NTM species and the involvement of organs are shown in Table 2.

**Table 2.** Frequencies of NTM species by disease sites.

Species distribution	Disseminated NTM (*n* = 43)	NTM pulmonary disease (*n* = 93)	Extrapulmonary NTM disease^a^ (*n* = 11)	Total (%) (*n* = 147)
SGM	26 (17.7)	59 (40.1)	6 (4.1)	91 (61.9)
*M. avium complex*	17 (11.6)	49 (33.3)	5 (3.4)	71 (48.3)
*M. avium*	1 (0.7)	12 (8.2)	1 (0.7)	14 (9.5)
*M. intracellulare*	13 (8.8)	37 (25.2)	4 (2.7)	54 (36.7)
*M. chimaera*	1^b^ (0.7)			1 (0.7)
*M. marseillense*	2^b^ (1.4)			2 (1.4)
*M. kansasii*	6 (4.1)	3 (2.0)		9 (6.1)
*M. gordonae*		4 (2.7)		4 (2.7)
others	3^c^ (2.0)	3^d^ (2.0)	2^e^ (1.4)	8 (5.4)
RGM	6 (4.1)	28 (19.0)	5 (3.4)	39 (26.5)
*M. abscessus*	6 (4.1)	21 (14.3)	4 (2.7)	31 (21.1)
*M. fortuitum*		6 (4.1)		6 (4.1)
others		1^f^ (0.7)		1 (0.7)
multiple	11^g^ (7.5)	6^h^ (4.1)		17 (11.6)

Data are shown as *n* (%).

RGM, rapidly growing non-tuberculous mycobacteria; SGM, slowly growing non-tuberculous mycobacteria. ^a^Includes skin/soft tissue infection (*n* = 7), lymphadenitis (*n* = 3), arthritis (*n* = 1; M. intracellulare). ^b^*M.* *chimaera* and *M.* *marseillense* are members of *M.* *avium* complex but were separated for clarity. ^c^*M.* *marinum* (*n* = 1), *M.* *lentiflavum* (*n* = 1), *M.* *szulgai* (*n* = 1). ^d^*M.* *paragordonae* (*n* = 1), *M.* *lentiflavum* (*n* = 1), *M.* *xenopi* (*n* = 1). ^e^*M.* *senegalense* (*n* = 1), *M.* *marinum* (*n* = 1). ^f^*M.* *monacense* (*n* = 1). ^g^*M.* *paraseoulense* and *M.* *gordonae* (*n* = 1), *M.* *colombiense*, *M.* *abscessus*, and *M.* *intracellulare* (*n* = 1), *M.* *kansasii* and *M.* *intracellulare* (*n* = 2), *M.* *marseillense* and *M.* *simiae* (*n* = 1), *M.* *avium* and *M.* *fortuitum* (*n* = 1), *M.* *avium* and *M.* *intracellulare* (*n* = 5). ^h^*M.* *avium* and *M.* *intracellulare* (*n* = 6).

### Organs involved and sources of specimens

**Figure 2.**
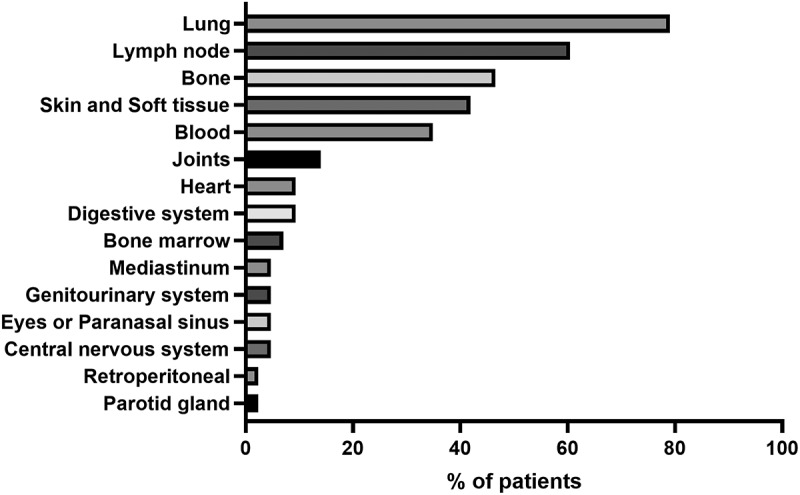
Sites of infections at presentation in the study subjects. NTM, such as those found in DNTM, could be hematogenously disseminated and form lesions in multiple organs.

NTM species were isolated from different parts of the body. In the 147 patients, the most common specimens were isolated from expectorated sputum samples (*n* = 48; 32.3%), followed by bronchial washing or bronchoalveolar lavage (*n* = 47; 32%). Other common specimens obtained from normally sterile sites included blood culture (*n* = 18; 12.2%), lymph nodes (*n* = 12; 8.2%), skin and soft tissues (*n* = 8; 5.4%), pulmonary biopsy (*n* = 7; 4.8%), synovial fluid (*n* = 4; 2.7%), bone tissue (*n* = 3; 2.0%), pus cultures (*n* = 3; 2.0%), and pleural fluid (*n* = 3; 2.0%). Among the 43 cases of disseminated infection, the most commonly involved organ was the lung (79.07%), followed by the lymph nodes (60.47%) (Figure 2).

### Characteristics of the pathogenic microorganisms

**Figure 3.**
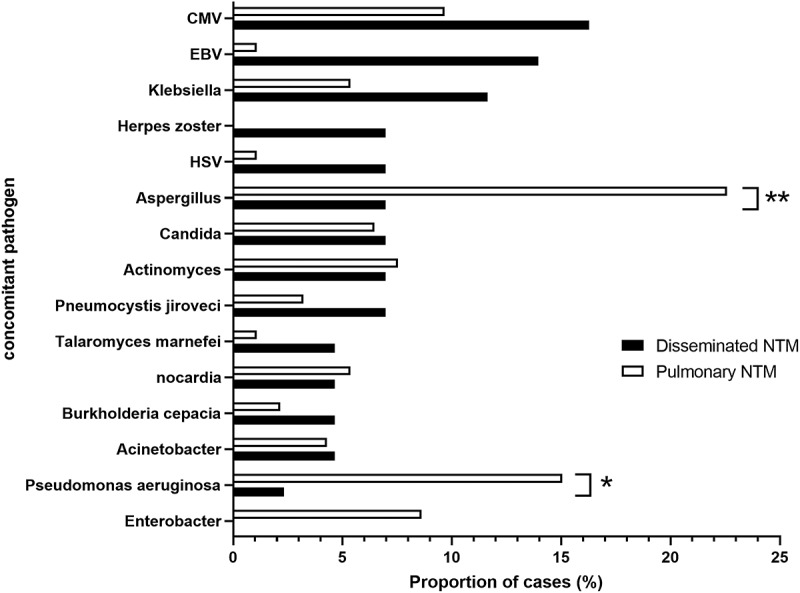
Distribution of pathogens in the lower respiratory tract, blood, and sterile area specimens from DNTM and NTM pulmonary disease patients. The top 15 coinfected pathogens in patients with NTM disease were identified. CMV, cytomegalovirus; EBV, Epstein–Barr virus; HSV, herpes simplex virus.

We conducted a series of analyses based on the microorganism characteristics. In the 147 patients, the most common types of coinfected pathogens were bacteria (48.3%), followed by fungi (21.8%) and viruses (14.3%). The incidence of *Aspergillus* and *P. aeruginosa* infection was significantly higher in the NTM pulmonary disease group than in the DNTM group, and both pathogens were isolated from the lower respiratory tract (Figure 3). In the extrapulmonary NTM group, only one case had active Epstein-Barr virus (EBV) infection. Detected pathogens were based on microbial types and considered pathogenic. NTM was not taken into consideration when cases were attributed to certain categories.

### Clinical outcomes, prognosis, and overall survival

We compared the clinical characteristics and laboratory test findings between the 36 patients who died and the 111 patients who survived during the follow-up period (Tables 1 and 3). The laboratory tests were conducted during the diagnosis of the disease. The median survival time from diagnosis to death was 29.4 (range, 0–105) months. The overall all-cause mortality rate was 10.1 per 100 person-years. Cumulative mortality was 14.9% at 1 year, 16.3% at 2 years, and 24.49% at 5 years in the total patients. Nineteen (52.8%) patients were diagnosed with disseminated infection in the non-survivor group, compared with only 24 (21.6%) in the survivor group. A significant association was found between disseminated infection and mortality (*p* < 0.001).

**Table 3.** Immune evaluation of the non-HIV patients with NTM disease.

indicators, median [IQR]	Overall (*n* = 147)	Survival (*n* = 111)	Death(*n* = 36)	*P*
WBC × 10^9^/L	7.12(4.84–11.26)	7.09(4.92–10.98)	7.64(4.19–12.38)	0.973
Neutrophils, × 10^9^/L	5.25(3.23–10.18)	5.13(3.21–10.25)	6.93(3.44–11.31)	0.627
Lymphocytes, × 10^9^/L	1.36(0.77–1.87)	1.47(1.09–2.02)	0.51(0.24–1.28)	0.000
Monocytes, × 10^9^/L	0.41(0.23–0.67)	0.43(0.29–0.71)	0.22(0.12–0.48)	0.001
NMLR	2.97(1.80–6.31)	2.45(1.63–4.96)	7.26(2.83–14.55)	0.000
NLR	4.17(2.21–9.64)	3.28(2.05–7.29)	10.06(4.78–27.40)	0.000
PLR	185.21(119.66–280.34)	168.55(116.34–44.16)	271.4(137.19–475.01)	0.005
CRP, mg/L	40.00(5.74–79.56)	18.64(3.43–63.56)	72(34.58–121.09)	0.056
CD3^+^T Lymphocyte*	863.00(422.50–1175.5)	934.50(668.25–1236.75)	427.00(288.00–826.50)	0.000
CD4^+^T lymphocyte*	433.00(174.00–673.50)	486.00(301.00–729.50)	172.00(88.50–460.50)	0.000
CD8^+^T lymphocyte*	301.00(172.00–478.50)	327.00(205.50–537.00)	193.00(140.00–328.50)	0.002
NK lymphocyte*	133.50(50.25–246.25)	177.00(81.00–275.28)	51.00(19.00–134.00)	0.000
B lymphocyte*	60.50(23.50–146.00)	69.00(42.00–165.00)	25.00(4.00–25.00)	0.007
CD4/CD8*	1.36(0.77–2.01)	1.36(0.87–2.02)	1.31(0.60–1.79)	0.312

NTM, non-tuberculous mycobacteria; IQR, interquartile range; WBC, white blood cells; NMLR, neutrophil-to-monocyte-plus-lymphocyte ratio; NLR, neutrophil-to-lymphocyte ratio; PLR, platelet-to-lymphocyte ratio; CRP, C-reactive protein; NK, natural killer. *Lymphocyte subsets (*n* = 91, mL).

Among the 36 patients who died, 29 (80.6%) received glucocorticoid or immunosuppressive treatment for different reasons: 9 for autoimmune diseases/connective tissue disorders, 3 for solid malignancies, 11 for haematological diseases, 2 for multiple diseases, 1 for nephrotic syndrome, and 3 for other diseases. Regarding the patients with multiple diseases, one had systemic sclerosis and colorectal cancer and the other had connective tissue disease and myelofibrosis. Among the 29 patients, corticosteroids were the most frequently used medications (*n = 1*8; 62.1%), followed by chemotherapy drugs (*n =* 8; 27.6%), cyclophosphamide (*n =*7; 24.1%), and mycophenolate mofetil (*n =* 5; 17.2%). Lymphocyte count was significantly lower in the patients who died than in the patients who survived, while the WBC and neutrophil counts did not differ between the groups. Clinical symptoms, including dyspnoea, showed no significant differences, except for fever. Neither immune defect disease nor underlying solid malignancy was associated with death, but the patients who died showed a higher proportion of haematological diseases and lower CD4^+^, CD8^+^, and B lymphocyte cell counts than the patients who survived. Of the 36 patients who died, 32 died of severe infection related to NTM, 1 died of gastrointestinal bleeding, and 1 died of cerebral haemorrhage. The remaining two patients had concomitant lymphoma and malignancy and these comorbidities may have contributed to their deaths.

### Effects of prognostic factors on the mortality

**Figure 4.**
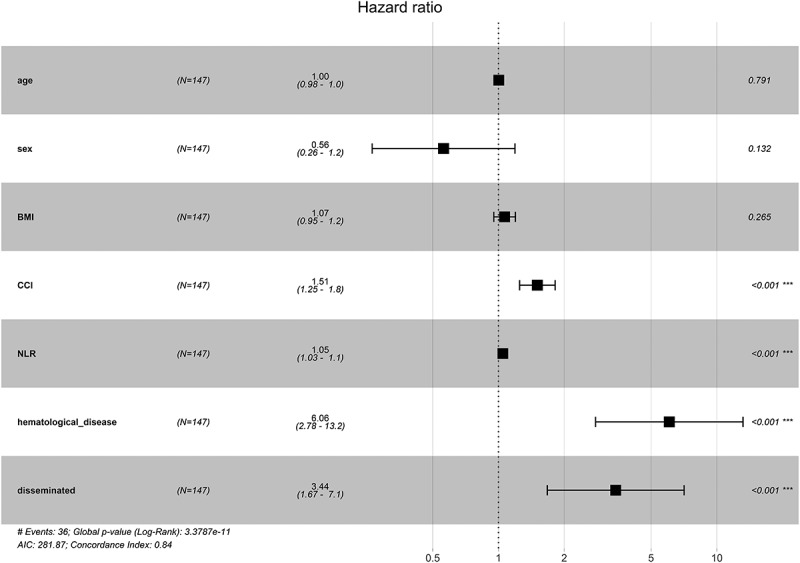
Forest plot showing the variables associated with mortality outcomes after multivariable Cox regression. CCI, Charlson Comorbidity Index; NLR, neutrophil-to-lymphocyte ratio.

**Figure 5.**
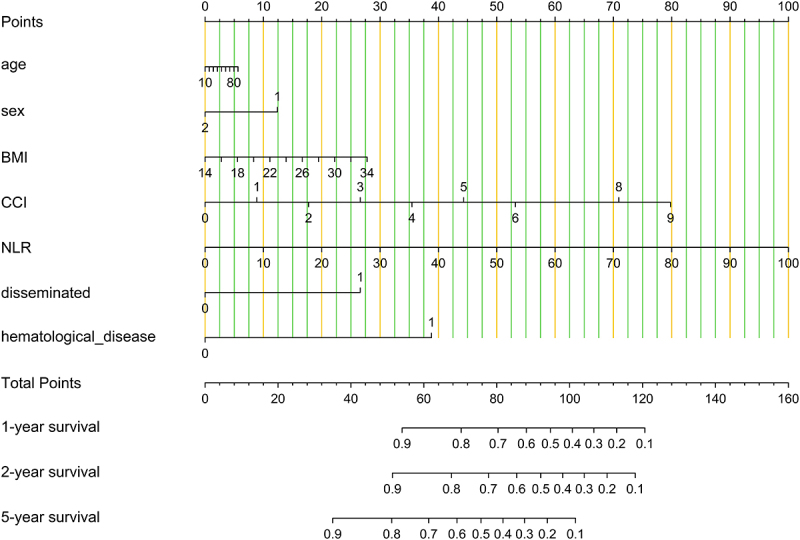
Nomogram based on the results of the multivariate Cox regression proportional hazards model. The line segment corresponding to each variable is marked with a scale representing the range of values available for that variable, while the length of the line segment reflects the size of the contribution of that factor to the end event. The total score is obtained by creating a line perpendicular to the point count axis at the position of the corresponding variable, and the individual scores for all variables are added together to give the total score. The bottom of the graph represents the corresponding survival rate. Calculation example of this nomogram: Total Points = 70-year-old (5 Points) + female (12.5 Points) +BMI 32 kg/cm^2^(25 Points) + CCI 2 (17.5 Points) + NLR 20 (20 Points) + no disseminated (0 Points) + no haematological disease (0 Points) = 80, probability for survival (1-year survival) = 0.7. CCI, Charlson Comorbidity Index; NLR, neutrophil-to-lymphocyte ratio.

**Figure 6.**
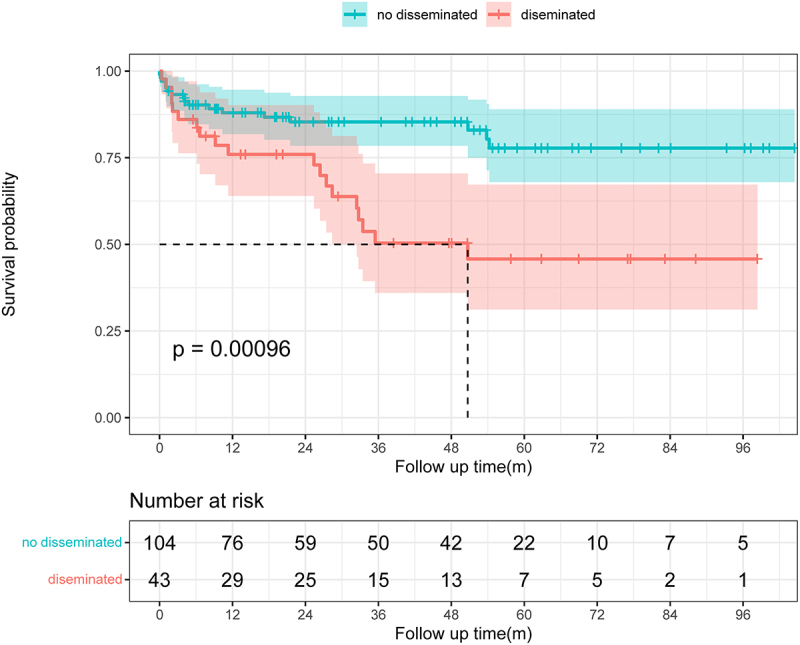
Kaplan–Meier survival curves from admission to 10 years of follow-up in the disseminated and non-disseminated disease groups. The survival rates were compared by the log-rank test.

**Figure 7.**
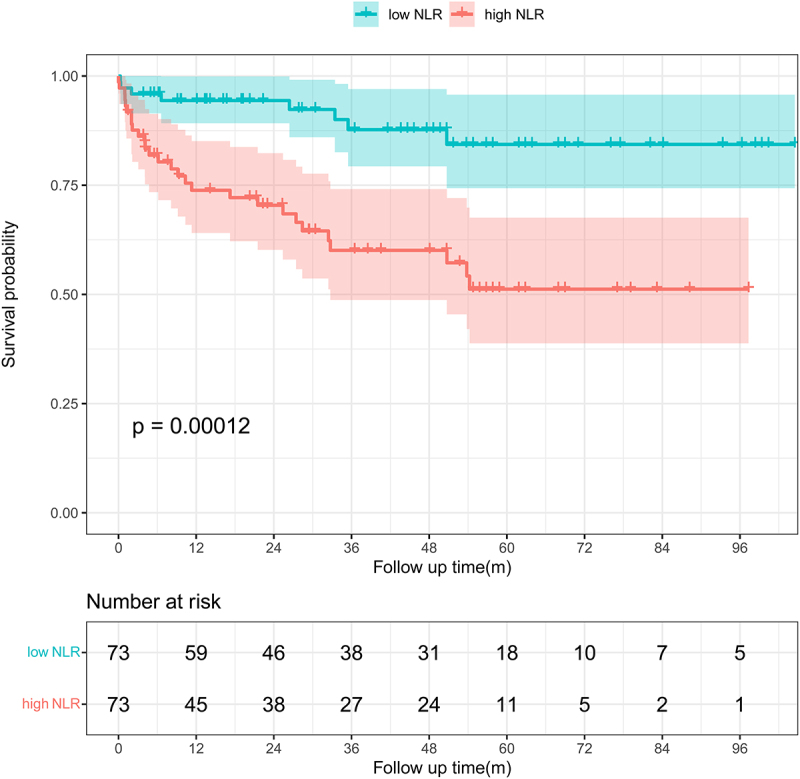
Kaplan–Meier survival curves from admission to 10 years of follow-up in the high and low NLR groups. The survival rates were compared by the log-rank test.

The impacts of different factors on mortality were assessed by univariate regression analyses. The results showed that sex, comorbidity, Charlson Comorbidity Index (CCI), glucocorticoid or immunosuppressive therapy, lymphocytes, NLR, NMLR, platelet-to-lymphocyte ratio (PLR), and disseminated infection were risk factors for mortality. Seven factors were subsequently included in a multivariate Cox proportional hazards regression model, after excluding factors that were collinear or had clinical relationships with other factors. We constructed a forest plot to show the variables associated with mortality to assess the ability of the prognostic scoring model to predict all-cause mortality (Figure 4). The results identified CCI, NLR, haematological disease, and disseminated infection as independent predictors of unfavourable outcomes. We generated a nomogram by weighting the score for each factor associated with the outcome based on the results of the multivariate Cox regression analysis (Figure 5). We used Kaplan – Meier survival curves to evaluate the prognostic factors associated with survival between the groups. The results showed that the risk of death was significantly increased in the disseminated infection group compared with the non-disseminated infection group (HR, 2.868; 95% CI, 1.489–5.524; *p* < 0.001) (Figure 6). The MAC and *M*. *abscessus* were the two species most frequently isolated. Compared with the MAC group, cumulative mortality in the *M. abscessus* group at 5 years was similar (37.9% vs. 32.1%). Subgroup analysis was then conducted. The *M. abscessus* had a trend of higher 5‐year cumulative mortality (39.5% vs.14.7%) in the NTM pulmonary disease subgroup, and lower 5‐year cumulative mortality (33.3% vs. 65.9%) in the DNTM disease subgroup, although these differences were not statistically significant. Using the median NLR value, the patients with NTM disease were divided into a high NLR group (>4.17) and a low NMLR group (≤4.17). The Kaplan – Meier analysis showed a significant difference in survival for the high NLR group versus the low NLR group (HR, 4.145; 95% CI, 1.89 − 9.10; *p* < 0.001) (Figure 7).

### Effects of NLR and NMLR on prediction of prognosis

**Figure 8.**
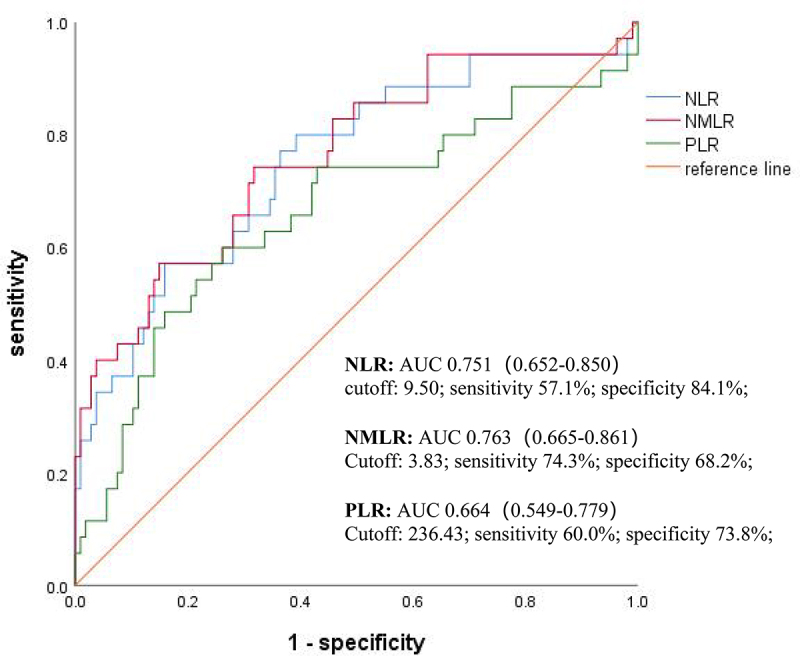
Discriminatory accuracy for prediction of mortality in patients with NTM disease using ROC curves and AUC values.

ROC curve analyses were performed to evaluate the predictive values of NLR, NMLR, and PLR. The area under the curve (AUC) values for NLR, NMLR, and PLR were 0.751, 0.763, and 0.664, respectively. Based on the ROC analysis, the optimal cut-off values for NLR, NMLR, and PLR were 9.50, 3.83, and 236.43, respectively (Figure 8).

## Discussion

NTM infection, as a group of opportunistic pathogens yet severe systemic infection, has emerged as an increasingly prevalent disease in HIV and non-HIV immunocompromised patients [20,21]. It has become an important pathogenic infection with a high mortality rate and an increasing incidence rate, particularly during the past decade. Disseminated infection is a rare complication in immunocompromised hosts. In this study, we investigated the risk factors associated with mortality and explored the clinical courses and outcomes of patients. The results showed that NLR and CCI were associated with poor prognosis and identified as independent risk factors for all-cause mortality in patients with NTM disease. Furthermore, we constructed a nomogram model to predict the long-term survival probability of patients with NTM disease. Patients with haematological diseases and disseminated infection had a significantly higher rate of all-cause mortality than patients without these factors.

The distribution of NTM species differs among regions [22,23]. In this study, the most frequent NTM species was MAC, followed by *M. abscessus*. Meanwhile, the predominant NTM species were MAC and *M. gordonae* worldwide, mainly from Europe [24]. The results also showed that SGM was identified as the dominant NTM species, compared to RGM. Yu et al. [7] reported a similar proportion of SGM in Northern China. Additionally, we identified two cases of *M. marseillense* infection in immunocompromised patients, one diagnosed with systemic lupus erythematosus and the other with anti-interferon-*γ* neutralizing autoantibody-associated immunodeficiency syndrome. We speculated that immunocompromised individuals might be more susceptible to *M. marseillense* infection. Notably, the incidence of *Aspergillus* and *P. aeruginosa* infection was significantly higher in the NTM pulmonary disease group than in the DNTM group, and the most common concomitant pathogens in the DNTM group were CMV and EBV. These findings suggest that patients with underlying lung structural diseases may be susceptible to coinfection with *Aspergillus* or *P. aeruginosa* and NTM, similar to those from previous studies [25]. NTM pulmonary disease often occurs in individuals with bronchiectasis, which provides a suitable microenvironment for the infection of these organisms. Patients with immune system disorders may be susceptible to the development of dissemination and coinfection with NTM and viruses. As described above, clinicians should pay more attention to possible infectious pathogens and strengthen the capacity for their detection.

The incidence of NTM infection, mainly pulmonary, has been increasing globally, and several studies have provided high overall estimates for case fatality rates in patients with NTM pulmonary disease [3]. Hu et al. [8] reported that the 5-year case fatality rate for people with HIV and NTM disease was 22.6%. A high mortality rate was also noted in this study. The high mortality rate may be due to the high number of patients with disseminated infection and the high proportion of immunocompromised hosts. The findings suggest that clinicians should pay more attention to NTM infection, especially in patients with haematological disease. Compared with the MAC, the *M. abscessus* had a trend of higher 5‐year cumulative mortality in the pulmonary NTM subgroup, similar to previous studies [26], although we could not demonstrate a significant difference, probably due to the limited numbers of patients. Abate G [26] reported that one-third of the patients with NTM pulmonary disease died in the *M. abscessus* group. Mori [27] showed that for NTM-related death, *M. abscessus* was a predictive factor for mortality in patients with NTM pulmonary disease. Song [28] showed that more than one-third of patients died in the MAC group, and no patients died in the *M. abscessus* group among renal transplant recipients. Another notable finding was that CCI, NLR, haematological disease and disseminated infection were identified as independent predictors of mortality in our multivariate Cox regression model, and we further generated a nomogram by weighting the score for each factor. Tsumura et al. [29] reported that active screening of NTM should be required in RGM-induced pulmonary disease in patients with pulmonary graft-versus-host disease, RGM bloodstream infection in patients with acute lymphoblastic leukaemia, and SGM infection in patients with inborn errors of immunity. NTM patients with these factors, especially those with haematological disease, should be recommended for closer monitoring and stronger combination treatment to improve their prognosis.

NLR is considered to be an index of inflammation and can be calculated by dividing neutrophil count by lymphocyte count. Severe inflammation may stimulate the production of neutrophils and accelerate the apoptosis of lymphocytes. NLR is emerging as a readily available biomarker for the estimation of mortality risk and disease severity. Previous studies indicated that NLR can be used as a biomarker to predict disease severity and mortality in patients with COVID-19 [30], and that high NLR may indicate an unfavourable prognosis in patients with sepsis [31]. Somboonviboon et al. [32] reported a patient with idiopathic CD4^+^ lymphocytopenia who presented with severe DNTM disease and indicated that it should be noted whether patients with isolated DNTM disease have a secondary immune deficiency. DNTM has been described in immunocompetent individuals as well as in those with inborn errors of immunity who may have an increased risk of infection [33]. GATA2 deficiency is also known as MonoMAC syndrome because of the typical features of monocytopenia and disseminated MAC [34,35]. Consistent with these reports, we identified NLR as an important independent risk factor for mortality in non-HIV patients with NTM disease. The ratio may be a very simple and easily obtainable clinical laboratory indicator for predicting disease prognosis and immune system disorders. As a biological indicator of systemic inflammation, NLR might reflect the host immunity status, and depletion of lymphocytes may indicate severe immune disorders in NTM disease. Thus, patients with high NLR may be prone to the development of disseminated infection, ultimately leading to a poor prognosis.

There are several limitations to our study. First, it was a retrospective study, and the data were collected from a single centre. Our hospital is one of the largest and top-ranked tertiary hospitals, providing treatment for many patients with immune diseases and other diseases. This may be one of the reasons for the high proportion of patients with immune disorders and the high mortality rate in patients with NTM disease. Second, some patients lacked species identification and were excluded from the study. The results can represent the situation in the local area. Finally, not all patients had a complete analysis of lymphocyte subsets. However, lymphocyte subsets were analysed in the majority of our patients to evaluate their immune status. We did not include the results of genetic analysis and some patients may have genetic defects. Future research should include prospective, multicenter randomized controlled studies on large populations to evaluate the prognosis.

## Conclusion

Patients with disseminated NTM disease have a higher mortality rate than those without disseminated infection. CCI, NLR, haematological disease, and disseminated infection were identified as independent predictors of mortality in non-HIV patients with NTM disease. Clinicians should provide close monitoring and strong combination therapy for these patients to avoid a poor prognosis.

## Data Availability

The Data generated during the study is available at the repository named “Clinical Analysis and Risk Factors Associated with Poor Prognosis in Nontuberculous Mycobacterial Infection” at https://doi.org/10.7910/DVN/DCBMQ8.
